# Combined mechanisms of neural firing rate homeostasis

**DOI:** 10.1007/s00422-018-0768-8

**Published:** 2018-06-28

**Authors:** Paul Miller, Jonathan Cannon

**Affiliations:** Department of Biology and Volen National Center for Complex Systems, MS013, Brandeis University, Waltham, MA 02454 USA

**Keywords:** Homeostasis, Integral feedback control, Stochastic, Integrator, Feedback, Control theory, Mutual information

## Abstract

Spikes in the membrane potential of neurons comprise the currency of information processing in the brain. The ability of neurons to convert any information present across their multiple inputs into a significant modification to the pattern of their emitted spikes depends on the rate at which they emit spikes. If the mean rate is near the neuron’s maximum, or if the rate is near zero, then changes in the inputs have minimal impact on the neuron’s firing rate. Therefore, a neuron needs to control its mean rate. Protocols that either dramatically increase or decrease a neuron’s firing rate lead to multiple compensatory changes that return the neuron’s mean rate toward its prior value. In this primer, first as a summary of our previous work (Cannon and Miller in J Neurophysiol 116(5):2004–2022, 2016; Cannon and Miller in J Math Neurosci 7(1):1, 2017), we describe the advantages and disadvantages of having more than one such control mechanism responding to the neuron’s firing rate. We suggest how problems of two, coexisting, potentially competing mechanisms can be overcome. Key requirements are: (1) the control be of a distribution of values, which the controlled variable achieves over a fast timescale compared to the timescale of the control system; (2) at least one of the control mechanisms be nonlinear; and (3) the two control systems are satisfied by a stable distribution or range of values that can be achieved by the variable. We show examples of functional control systems, including the previously studied integral feedback controller and new simulations of a “bang–bang” controller, that allow for compensation when inputs to the system change. Finally, we present new results describing how the underlying signal processing pathways would produce mechanisms of dual control, as opposed to a single mechanism with two outputs, and compare the responses of these systems to changes of input statistics.

## Introduction

Homeostasis is a common requirement for a diverse range of biological systems. From the biochemical to the organismal scale, biological processes operate near-optimally under some conditions, but under other conditions, they fail. These conditions—such as size, temperature, acidity—are typically under tight control. The control of these conditions is homeostatic, meaning that following perturbations that cause a deviation from a target value or range, biological pathways are engaged to counter that change and return the condition to equilibrium.

**Fig. 1:**
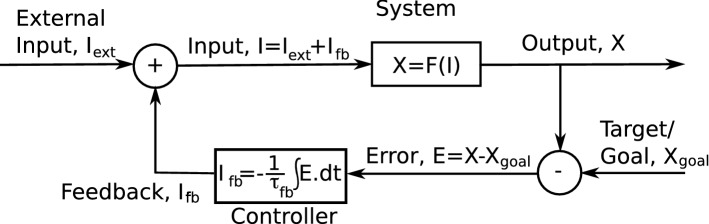
An integral feedback controller. In this example of integral feedback control, the output, *X*, changes transiently in response to changes in external input, *I*_ext_, but thereafter, the feedback signal, *I*_fb_, generated by the controller continuously changes the total input, *I*, until the output, *X*, reaches its target/goal value, *X*_goal_

Engineered systems often face similar homeostatic constraints to living systems in terms of the need to maintain properties of the system in a tight range. The field of control theory describes mathematically how such homeostasis is achieved. One of the simplest controllers is the integral feedback controller, which we will describe below as it is one of the canonical models of feedback control and because it includes the main processes likely to reside within any biological control system. In an integral feedback controller, any deviation from a set-point accumulates over time, and such accumulation is fed back to the system’s inputs in a subtractive manner. The circuit for integral feedback control is shown in Fig. 1, with the corresponding formalism in Eqs. 1–3.

The controller in Fig. 1 produces feedback, $I_{\text{fb}}$, by accumulating (integrating over time) the error signal:

1 $$ \tau_{\text{fb}} \frac{{{\text{d}}I_{\text{fb}} }}{{{\text{d}}t}} = - E = X_{\text{goal}} - X. $$

The feedback combines with the external input to the system to produce an output, $X$, according to:

2 $$ X = F\left( I \right) = F\left( {I_{\text{ext}} + I_{\text{fb}} } \right). $$

While these equations can be simulated easily, the control of the output, $X$, can be understood from Eq. 1, which tells us that the feedback will continuously change unless $X = X_{\text{goal}}$. Here, we assume the output increases with input, i.e., $F\left( I \right)$ is a monotonically increasing function, as will be the case when we consider the specific example of the output being a firing rate and the input being the total excitatory synaptic conductance of a neuron. In this case, the fixed point (i.e., the value of the variable at which the system no longer changes), $X = X_{\text{goal}}$, is stable because $\frac{{{\text{d}}X}}{{{\text{d}}t}} < 0$ when $X > X_{\text{tgt}}$ and $\frac{{{\text{d}}X}}{{{\text{d}}t}} > 0$ when $X < X_{\text{tgt}}$ (that is, any deviation causes it to change back to that value):

3 $$ \tau_{\text{fb}} \frac{{{\text{d}}X}}{{{\text{d}}t}} = \tau_{\text{fb}} \frac{{{\text{d}}I}}{{{\text{d}}t}}\frac{{{\text{d}}F}}{{{\text{d}}I}} = \frac{{{\text{d}}F}}{{{\text{d}}I}}\left( {X_{\text{goal}} - X} \right), $$

where we have substituted first from Eq. 2 then from Eq. 1.

### Homeostasis of neural firing rates

Neural firing rates are homeostatic, but in an unusual sense. Since variations in neural activity are necessary to convey information—and such conveyance of information is the raison d’être of neural activity—a homeostatic controller that enforced a fixed firing rate of a neuron would render the neuron useless. Rather, a homeostatic controller for neural activity might need to ensure the average rate, when sampled over a reasonable period of time, is maintained in a suitable range. Or, even more preferably, the controller might need to ensure the neuron’s firing rate varies over a particular range in order to transmit useful information.

Such behavior is in contrast to typical homeostatic controllers, for which any deviation from the mean is undesirable. For example, strong variability in body temperature would be disastrous for most warm-blooded animals, even if the mean temperature were maintained. Therefore, we might expect the control system for neural firing rate to have different properties from that of many other controllers—in particular, the timescale of feedback can (and should) be a lot slower than the timescale of variation in firing rates. See the work of O’Leary and Wyllie (2011) for a more thorough introduction to the use of control theory for homeostasis of cellular processes.

Some of the first measurements of homeostasis in neural systems indicated that the conductance of excitatory synapses scaled up or down in compensation when neural firing rates were artificially lowered or raised from their baseline levels (Turrigiano et al. 1998; O’Leary et al. 2010). We can represent such compensation as an integral feedback control process in which the feedback multiplicatively scales the inputs (rather than adds to or subtracts from the input as in standard controllers—see Fig. 1). The corresponding equations for a simplified model of neural firing rate, $r$, are:

4 $$ \tau_{r} \frac{{{\text{d}}r}}{{{\text{d}}t}} = - r + F\left[ {g_{\text{E}} S_{\text{E}} \left( t \right) + g_{\text{I}} S_{\text{I}} \left( t \right) - T} \right] $$

and

5 $$ \tau_{g} \frac{{{\text{d}}g_{\text{E}} }}{{{\text{d}}t}} = r_{\text{goal}} - r. $$

Equation 4 indicates how the neuron’s firing rate depends on time-varying excitatory inputs, $S_{\text{E}} \left( t \right)$, and inhibitory inputs, $S_{\text{I}} \left( t \right)$, via excitatory and inhibitory synaptic conductance, $g_{\text{E}}$ and $g_{\text{I}}$, respectively. The variable, $T$, represents a threshold—the amount of input the neuron requires before significant activity. In response to changes in input, a neuron’s firing rate changes very rapidly, over a timescale on the order of 10 ms, so the time constant $\tau_{r}$, is short and of this order. On the other hand, the homeostatic response described by Eq. 5 is much slower, with changes in synaptic conductance due to a neuron’s firing rate alone requiring at least tens of minutes and probably several hours to reach measurable levels. Therefore, the time constant, $\tau_{g}$, for these homeostatic changes is much larger than $\tau_{r}$. Equation 5 then represents an integration of the error signal, where the target rate, $r_{\text{tgt}}$, represents a fixed point of the system. If the synaptic inputs vary only slowly compared to $\tau_{g}$, then the system will reach the fixed point with $r = r_{\text{tgt}}$. When the synaptic inputs are more rapidly varying—as they are in vivo—then the changes in excitatory conductance, $g_{\text{E}}$, cannot counteract any rapid, transient change in firing rate, $r$. However, the conductance will, for example, increase more than it will decrease over time if the rate fluctuates more often, or to a greater extent, below rather than above the target rate. In this manner, Eq. 5 leads to a control of the mean of the firing rate, $r$.

## The benefit of multiple control mechanisms

A single controller can control a single variable or, more precisely, a single function (such as the mean) of that variable when it fluctuates. Control of a neuron’s mean firing rate is important, in simplest terms because each action potential produced by a neuron costs energy, so excessive activity is wasteful. On the other hand, a neuron that rarely or never produces action potentials is taking up resources to function as a cell but is of minimal value for information processing.

When we consider the number of successive feedforward connections and, even more importantly, the feedback connections, the need for a control system becomes paramount (Turrigiano and Nelson 2004). For example, an imbalance between excitatory and inhibitory feedback connections in the hippocampus can lead to hyperexcitability (Bateup et al. 2013) and possibly contributes to epilepsy (Badawy et al. 2012).

Yet, as well as maintaining a satisfactory mean level of activity, neurons must also vary their firing rates to perform any useful information processing. One can think of neurons mapping a range of inputs into a range of outputs according to their firing rate response curves. These curves have particular regions over which changes in input cause significant changes in output. Control of the mean firing rate of a neuron could ensure the mean input falls in the neuron’s responsive range. However, it may be better to also match the range of inputs to the entire responsive range of the neuron, so that any change in input leads to a change in output. To achieve such responsiveness, a neuron would need to control the variance of its firing rate, which—as we will see in Sect. 3—requires cooperation of two controllers (Triesch 2007). Here we focus on sensory systems, in which the input statistics may not be known a priori and a neuron's spikes should contain some useful information about the current input.

### Mutual information

In order to quantify the above argument, we chose to measure the mutual information between a simulated neuron’s input and its output when additional noise is included within the neuron. We selected ten equally spaced levels of input current, switching between these levels every 500 ms. By calculating the mean rate of the neuron and comparing that to the input current during each period, we assessed how well a simulated neuron distinguished between the different levels of input (for details see Cannon and Miller 2016).

**Fig. 2:**
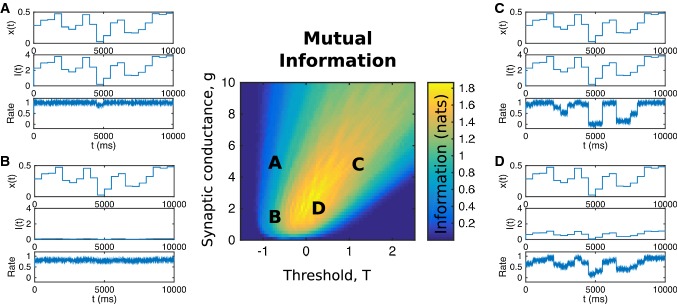
Maximization of mutual information between a neuron’s inputs and response requires tuning of two parameters. **A**–**D** The response, using four different parameter sets, of a firing rate model with sigmoidal output curves in response to synaptic inputs, $x\left( t \right)$, which change every 500 ms to produce input current, $I\left( t \right) = gx\left( t \right)$, and firing rate, $r\left( t \right) = 1/\left[ {1 + {\text{e}}^{T - I\left( t \right)} } \right] + \eta \left( t \right)$ where $\eta \left( t \right)$ is zero mean, white noise with standard deviation 0.1. **A** Low threshold and high gain result in a high firing rate that varies little with changes in synaptic input. **B** Low threshold and low gain produce an unvarying response at slightly lower rate. **C** High threshold and high gain produce a response which samples extreme high and low rates more than intermediate rates. **D** At the optimal threshold and gain, the neural response more faithfully follows changes in the inputs to produce greatest mutual information. Center: Mutual information (MI) as a function of threshold and synaptic strength has a broad optimum that nevertheless requires control of the two parameters. Values of MI are given by the color scale and measured in natural logarithm units, or nats, rather than bits. This figure is based on Fig. 4 of our earlier work (Cannon and Miller 2016)

In Fig. 2, we show examples of the neural responses to such current steps when the synaptic input gain and firing threshold of the neuron are set to different values. If the neuron is too excitable (upper left) or unexcitable (lower right) its rate varies little from its maximum saturated value or from zero, respectively, and information transfer is negligible. However, even under conditions where the mean rate is reasonable, if synaptic conductance is too small (lower left), then the neuron is unresponsive. Moreover, if synaptic conductance is too high (upper right), the neuron has only two responses—inactive or maximally active—to inputs of different levels, so the mutual information is not as high as at its optimum (point D) where intermediate levels of input can produce differential responses.

While the results presented here are produced with a firing rate model neuron (Eq. 4), similar behavior arises from a voltage-based spiking-neuron model (Cannon and Miller 2016).

### Tuning an integrator in a neural circuit

Mutual information simply indicates how much information about an input can be obtained from an output, so is really a measure of information transfer. In the brain, however, neural circuits must do more than transfer information in their sensory inputs. Rather, brains must allow us to combine separate sources of information over multiple timescales and calculate a desired response. Of the many computations required for such neural processing, one that is particularly amenable to study in the context of fine-tuning is the integration of inputs across time.

Neural integrators have been studied in the context of gaze control (Goldman et al. 2002, 2003; Seung 1996; Seung et al. 2000a), short-term memory of a continuous quantity (called parametric working memory) (Romo et al. 1999; Miller et al. 2003; Machens et al. 2005; Miller and Wang 2006), and decision making (Huk and Shadlen 2005; Miller and Katz 2010, 2013; Wong et al. 2007; Wong and Wang 2006; Wang 2002, 2008). An important property of an integrator is that transient inputs shift the activity of the integrator (up or down depending on whether the input is positive or negative) but the history of inputs accumulates and remains when the input is removed.

Since neural activity decays on a rapid timescale (for example the time constant due to a neuron’s membrane capacitance is on the order of 10 ms) one question has been how to extend the timescale of information decay by up to 100-fold so that behavior on the one-second timescale can be explained. In a neural circuit with excitatory feedback, the timescale can be increased by a factor that depends on the extent of fine-tuning (Seung et al. 2000a, b). For example, if we assume a linear model of neural activity and feedback:

6 $$ \tau_{r} \frac{{{\text{d}}r}}{{{\text{d}}t}} = - r + g_{\text{E}} \cdot S_{\text{E}} - T $$

with synaptic input dependent on the presynaptic firing rate of the same cells as well as external input, $S_{\text{E}} = \alpha r + S_{\text{in}}$, then the dynamics can be rewritten as (Seung 1996; Seung et al. 2000b)

7 $$ \frac{{{\text{d}}r}}{{{\text{d}}t}} = - \frac{r}{{\tau_{r} /\left( {1 - \alpha g_{\text{E}} } \right)}} + \frac{{g_{\text{E}} S_{\text{in}} - T}}{{\tau_{r} }}. $$

The effective time constant in Eq. 7 is $\tau_{r} /\left( {1 - \alpha g_{\text{E}} } \right)$, which extends to infinity in the limit $\alpha g_{\text{E}} = 1$. When $\alpha g_{\text{E}} < 1$, the firing rate converge to a fixed point, and when $\alpha g_{\text{E}} > 1$, the firing rate increases without bound. In the limit of $\alpha g_{\text{E}} = 1$, the firing rate will represent a perfect integration of the input, $S_{\text{in}}$, so long as the threshold is also controlled, such that $T = 0$. That is, in the space of possible values of $g_{\text{E}}$ and $T$, only a single point corresponds to a perfect integrator, so a dual-control system would be essential for this point to be reached and integration produced in such a feedback circuit.

## The problem of competing controllers

**Fig. 3:**
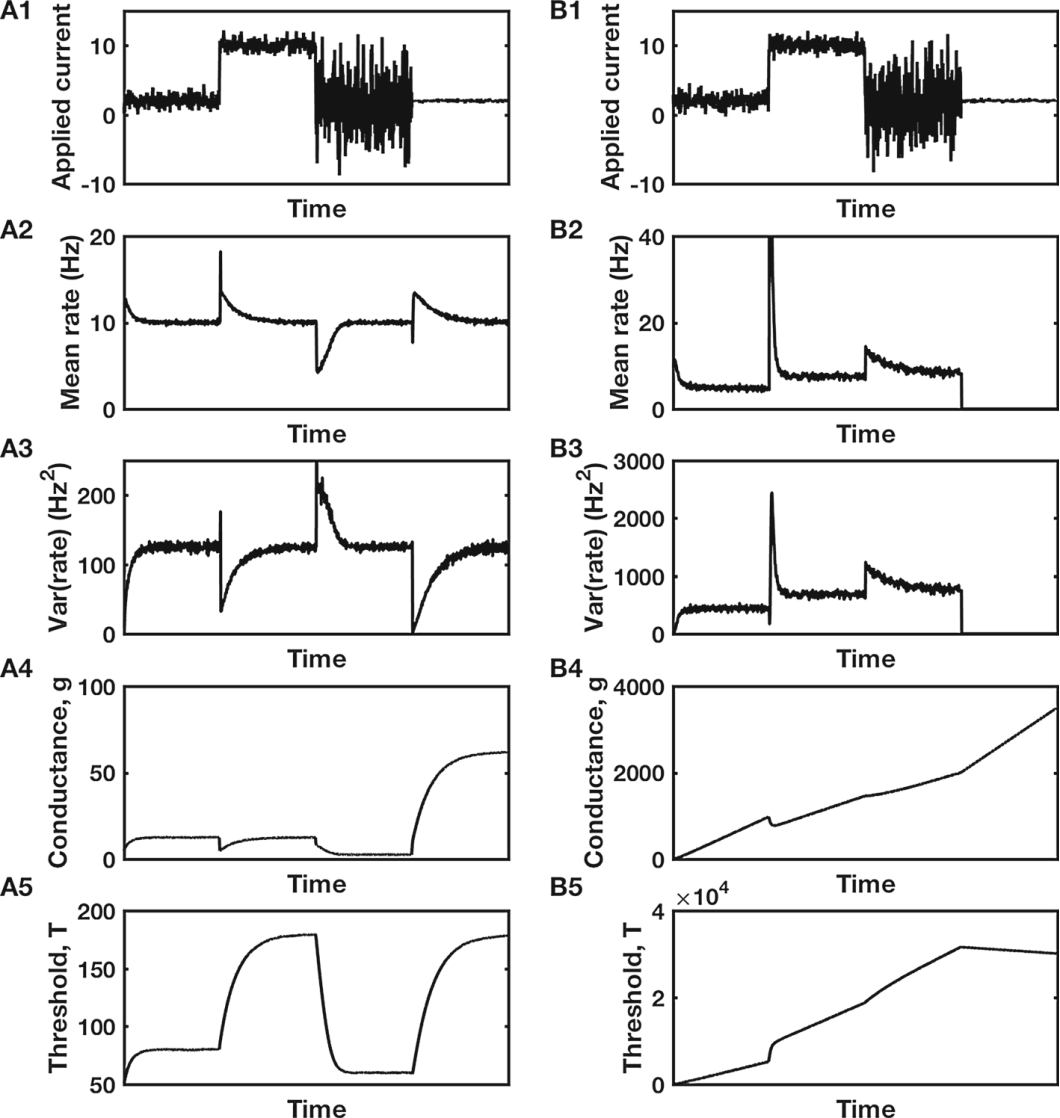
Cooperation or competition between two integral feedback controllers. **A** Simulation of Eqs. 4, 8, and 9, in a functioning dual-control system with $r_{{{\text{goal}}\left( T \right)}} = 10$ Hz, $r_{{{\text{goal}}\left( g \right)}} = 15$ Hz, $\tau_{T} = \tau_{g} = 500$ s, with sigmoidal firing rate curve, $F\left[ {g_{\text{E}} S_{\text{E}} \left( t \right) - T} \right] = F\left[ X \right] = \frac{{r_{ \hbox{max} } }}{{1 + { \exp }\left( { - X/\sigma } \right)}}$ with $r_{ \hbox{max} } = 100$ Hz and $\sigma = 20$. **B** An equivalent simulation, but with $r_{{{\text{goal}}\left( T \right)}} = 15$ Hz and $r_{{{\text{goal}}\left( g \right)}} = 10$ Hz, while all other parameters are unchanged, produces wind-up to non-physiological levels. **A1**, **B1** Input current that transitions between four different distributions. **A2**, **B2** Mean firing rate and **A3**, **B3** variance in firing rate in response to the inputs of **A1**, **B1**. **A4**, **B4** Compensatory changes in synaptic conductance and **A5**, **B5** intrinsic threshold of the neuron. Notice in **B4** and **B5** that neither conductance nor threshold ever stabilize, so depict the phenomenon of wind-up. Code used to produce this figure is available as quad_lin_control.m

When two controllers monitor the same signal, a problem of competition can arise. Each controller has its own set-point. If the signal is fixed at the set-point of one controller, it will produce a constant error signal to the second controller, causing the second controller to ramp up its feedback until the error for the second controller is removed. However, as the second controller ramps up its feedback so as to shift the signal, the first controller receives an error signal and ramps up its own feedback so as to return the signal to its own set-point. Such a competitive tug-o-war between the two controllers produces “wind-up” (see Figs. 3B4, B5, and 4B4, B5), whereby each controller’s feedback increases more and more until some biophysical maximum is reached (not depicted in the figure panels) and the system no longer acts as an integral feedback controller. Such a situation is manifestly undesirable.

**Fig. 4:**
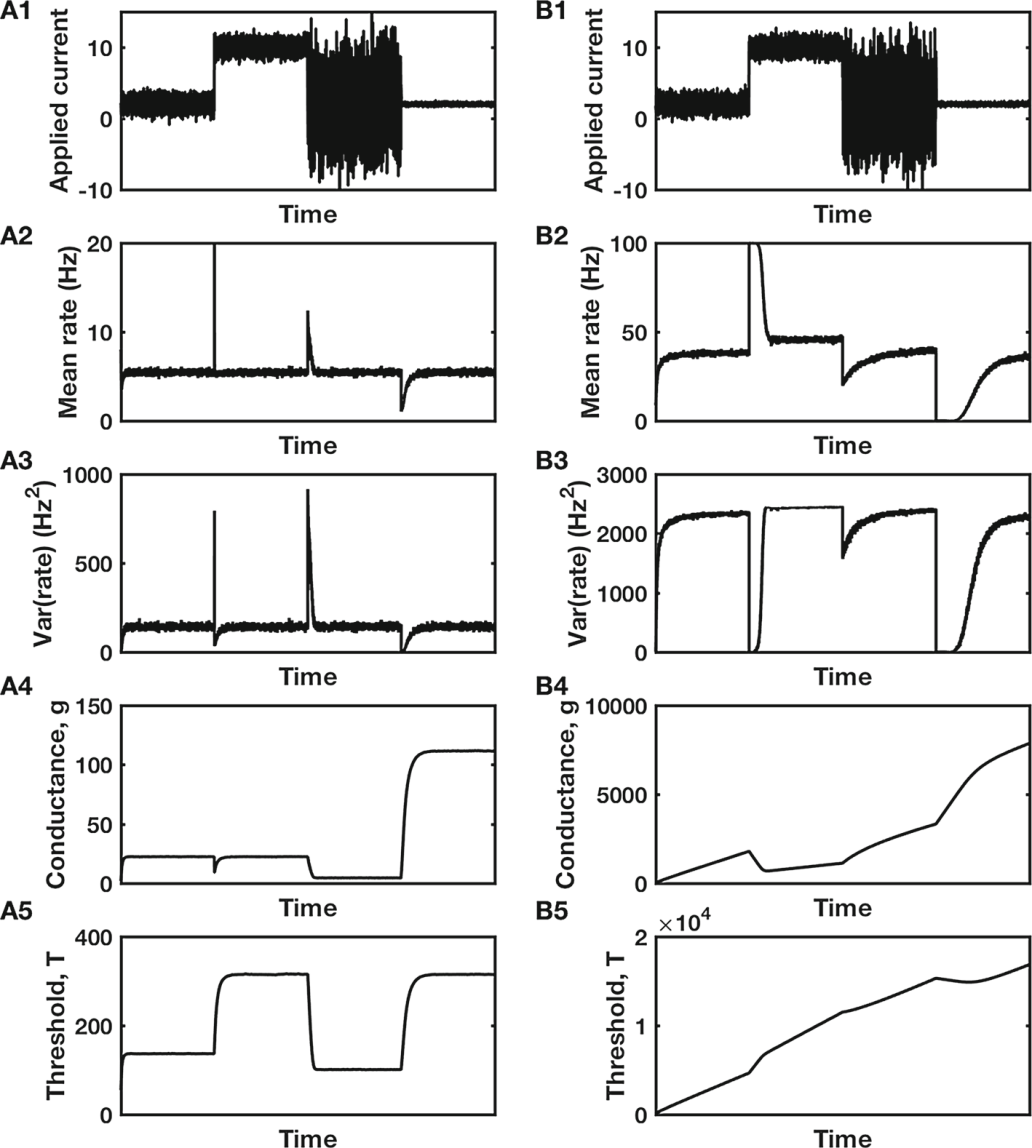
Firing rate control by a dual bang–bang controller. **A** Mean and variance of firing rate recover to original values following changes in inputs and stable compensatory changes in conductance and threshold. Parameters are $r_{{{\text{step}}\left( T \right)}} = 1$, $r_{{{\text{step}}\left( g \right)}} = 15$, $p_{T} = 0.5$, $p_{g} = 0.1$. **B** Wind-up in a similar system—while rate appears to be controlled, the conductance and threshold are continually increasing, to non-physiological levels. Parameters are $r_{{{\text{step}}\left( T \right)}} = 1$, $r_{{{\text{step}}\left( g \right)}} = 15$, $p_{T} = 0.1$, $p_{g} = 0.5$. **A1**, **B1** Input current that transitions between four different distributions. **A2**, **B2** Mean firing rate and **A3, B3** variance in firing rate in response to the inputs of **A1**, **B1**. **A4**, **B4** Compensatory changes in synaptic conductance and **A5**, **B5** intrinsic threshold of the neuron. Notice in **B4** and **B5** that neither conductance nor threshold ever stabilize, so depict the phenomenon of wind-up. Firing rate model of the neuron is identical to that of Fig. 3. Code used to produce this figure is available as dual_bang_bang.m

However, the problem of wind-up can be avoided if certain conditions hold. Two necessary conditions are: (1) The signal fluctuates on a rapid timescale compared to the timescale of feedback control, and (2) feedback from the controllers is based on different functions of the signal, at most one of which is linear. The first condition allows the controllers to act upon the distribution of values attained by the signal, rather than a single value. The second condition ensures the controllers are fixing different moments or different combinations of moments of that distribution (see also Triesch 2007). We will show later that two additional conditions must be satisfied when there are two controllers, one to ensure that the target distribution has nonnegative moments and the other to ensure the targets correspond to stable rather than unstable fixed points (Cannon and Miller 2016, 2017).

### A simple dual-control model

To understand how two controllers can cooperate, consider a system with one controller whose feedback is based on the integral of the signal and a second controller whose feedback is based on the integral of the square of the signal (Triesch 2007). For example, when neural firing rate is controlled, these controllers would respond according to:

8 $$ \tau_{T} \frac{{{\text{d}}T}}{{{\text{d}}t}} = r - r_{{{\text{goal}}\left( T \right)}} $$

and

9 $$ \tau_{g} \frac{{{\text{d}}g_{\text{E}} }}{{{\text{d}}t}} = r_{{{\text{goal}}\left( g \right)}}^{2} - r^{2} . $$

The first controller (Eq. 8) adjusts the neuron’s threshold to ensure its mean firing rate satisfies $<r>\,= r_{{{\text{goal}}\left( T \right)}}$. In practice, such control of intrinsic excitability (Desai et al. 1999) could be based on adjustment of the number of sodium and/or potassium channels implanted in the membrane of the soma near the axon hillock where spikes are generated. The second controller (Eq. 9) adjusts synaptic conductance to ensure that the neuron’s mean-squared firing rate satisfies $<r^{2}>\,= r_{{{\text{goal}}\left( g \right)}}^{2}$. In practice, such control could be based on adjustment of the number or type of glutamate receptors in the postsynaptic density.

By combining Eqs. 8 and 9, we see that both the mean and the variance of the firing rate are set by such a dual-control system, with ${\text{Var}}\left( r \right) = \, <r^{2}>- <r^{2}> \,= r_{{{\text{goal}}\left( g \right)}}^{2} - r_{{{\text{goal}}\left( T \right)}}^{2}$. Since the variance of any distribution must be positive, we straight away see the condition on the two target rates, $r_{{{\text{goal}}\left( g \right)}}^{2} > r_{{{\text{goal}}\left( T \right)}}^{2}$, or (since any rate must be positive) $r_{{{\text{goal}}\left( g \right)}} > r_{{{\text{goal}}\left( T \right)}}$ (see Fig. 3).

At first sight, an alternative control system is possible, with $\frac{{{\text{d}}g_{\text{E}} }}{{{\text{d}}t}}$ linearly dependent on the rate and $\frac{{{\text{d}}T}}{{{\text{d}}t}}$ following a quadratic dependence, so long as the opposite condition is satisfied, $r_{{{\text{goal}}\left( T \right)}} > r_{{{\text{goal}}\left( g \right)}} .$ Indeed, such a control system with the synaptic conductance and threshold controllers flipped, does possess a fixed point with allowable firing rate variance greater than zero. However, the flipped control system turns out to be unstable. The instability arises because synaptic conductance scales the variance of firing rates while the threshold scales only the mean (This can be seen from Eq. 6, where the inputs are multiplied by $g_{\text{E}}$ and a multiplicative factor changes the variance, while the threshold is subtracted so has no impact on the variance). In a situation in which the firing rate variance is too high, but the mean is too low, the neuron should decrease the synaptic conductance and decrease the threshold to approach the fixed point determined by the target rates. However, in the flipped system, if the mean rate is too low then synaptic conductance would increase, while the variance being too high might cause a concomitant increase in threshold. Therefore, the flipped controller adjusts the system’s parameters in the opposite direction needed to reach the fixed point. Simulations and mathematical analysis of the stability of fixed points verify this intuition (Cannon and Miller 2017).

The results from the combined linear and quadratic controllers can be generalized to arbitrary control functions:

10 $$ \tau_{T} \frac{{{\text{d}}T}}{{{\text{d}}t}} = f_{T} \left( r \right) - f_{T} \left( {r_{{{\text{goal}}\left( T \right)}} } \right) $$

and

11 $$ \tau_{g} \frac{{{\text{d}}g_{\text{E}} }}{{{\text{d}}t}} = f_{g} \left( {r_{{{\text{goal}}\left( g \right)}} } \right) - f_{g} \left( r \right). $$

To avoid the need for fine-tuning, the two control functions, $f_{T}$ and $f_{g}$, should have different curvatures at their target rates, in which case the function with greater curvature (strictly its second derivative divided by its first derivative) should be $f_{g}$, the one for the synaptic input controller (Cannon and Miller 2017).

Indeed, we were able to show that a control system with these constraints and appropriate target/goal rates could, from arbitrary initial conditions, adapt its threshold and synaptic input conductance so as to maximize mutual information between a neuron’s activity and its inputs, and to optimize performance of a neural integrator (Cannon and Miller 2016). More importantly, following any change in input statistics, or in the case of the integrator circuit, loss of 20% of the neurons providing feedback, the control system led to recovery of optimal function.

### A “bang–bang” controller

Another example of a feasible system of two controllers is one in which the controllers respond to different step functions of the signal:

12 $$ \tau_{T} \frac{{{\text{d}}T}}{{{\text{d}}t}} = - p_{T} + H\left( {r - r_{{{\text{step}}\left( T \right)}} } \right) $$

and

13 $$ \tau_{g} \frac{{{\text{d}}g_{\text{E}} }}{{{\text{d}}t}} = p_{g} - H\left( {r - r_{{{\text{step}}\left( g \right)}} } \right) $$

where $H\left( x \right)$ represents the Heaviside step-function, $H\left( x \right) = 1$ if $x \ge 0$ and $H\left( x \right) = 0$ if $x < 0$.

Such controllers with binary outputs are known as “bang–bang” controllers. Each controller would be ensuring that the neuron spends a certain fraction or proportion of its time, $p_{T}$ and $p_{g}$, with its firing rate above that controller’s step-point, respectively $r_{{{\text{step}}\left( T \right)}}$ and $r_{{{\text{step}}\left( g \right)}}$.

For two bang–bang controllers to cooperate rather than compete, the target fraction of time above the higher step-point must be less than the fraction above the lower step-point (see Fig. 4). Also, as in the previous example, in order for a system with step-controllers to approach a stable equilibrium, the controller with the higher step-point should be the one that scales the synaptic conductance rather than the threshold. That is, $r_{{{\text{step}}\left( g \right)}} > r_{{{\text{step}}\left( T \right)}}$ and $p_{T} > p_{g}$. Indeed, if we simulate the system with $r_{{{\text{step}}\left( g \right)}} < r_{{{\text{step}}\left( T \right)}}$, the synaptic input conductance rapidly decreases to zero and the neuron becomes unresponsive.

While a system of dual bang–bang controllers can be more robust, they have the disadvantage of lacking a stable fixed point. For example, from Eq. 13, the excitatory synaptic conductance is either increasing at a constant rate of $p_{g} /\tau_{g}$ when $r < r_{{{\text{step}}\left( g \right)}}$ or decreasing at a constant rate of $\left( {1 - p_{g} } \right)/\tau_{g}$ when $r > r_{{{\text{step}}\left( g \right)}}$. For many control systems, the consequent alternation of switching on then switching off of different systems is undesirable. However, a living cell such as a neuron is a dynamic system whose ion channels and receptors—which determine its synaptic conductance—are mobile, being replaced and manufactured unceasingly. Therefore, a bang–bang controller, by simply switching between two distinct rates either of insertion or removal of membrane proteins, can be feasibly implemented with no additional “operational costs” to the cell (O’Leary et al. 2013, 2014).

## Underlying processes

### The need for a biochemical integrator

Engineered controllers include, in addition to integral controllers, proportional controllers and derivative controllers, in which the feedback signal is either proportional to the system’s output or to its derivative (O’Leary and Wyllie 2011). More commonly, combinations of the three are used. However, to be at all useful, the proportional and derivative controllers should respond rapidly compared to the timescale of signal variations—as does, for example, the rapid feedback from interneurons in many cortical circuits. The observed slower homeostatic control is more compatible with integral feedback control, which at heart requires a biochemical process within the neuron to integrate any error signal (Somvanshi et al. 2015).

A biochemical integrator requires a zeroth-order chemical reaction (O’Leary et al. 2013, 2014). That is, for any product state of the system to accumulate with the temporal integral of a reactant—i.e., the signal—the rate of decay of that product state should be independent of the amount accumulated. This can be seen by supposing the opposite was true, so that in general the rate of production of an accumulated biochemical product state, $P$, were some monotonic function, $F$, of an input signal, $S$, and the rate of loss of the accumulated product state were a monotonic function, $G$, of that accumulated product state:

14 $$ \frac{{{\text{d}}P}}{{{\text{d}}t}} = F\left( S \right) - G\left( P \right) $$

then the steady state would be given by $P = G^{ - 1} \left[ {F\left( S \right)} \right]$, that is a single, fixed value of $P$ results from any given value of $S$ and no temporal accumulation occurs. Such a response would be an example of proportional control.

However, if instead the rate of loss of the accumulated state is constant (at least independent of $P$) then we have

15 $$ \frac{{{\text{d}}P}}{{{\text{d}}t}} = F\left( S \right) - C $$

and the amount of $P$ is given by a temporal integral:

16 $$ P = \int \left[ {F\left( S \right) - C} \right]{\text{d}}t $$

whose steady state requires control of the input signal at $S = F^{ - 1} \left( C \right)$. Therefore, as long as increased accumulation of $P$ leads to a reduction in $F\left( S \right)$, then integral feedback control is achieved.

Rate of loss of $P$ could be independent of $P$ if the amount of $P$ saturates an enzyme or any other degradation system that can cause $P$ to decrease. The Michaelis–Menten reaction scheme is an example of such saturating enzymatic kinetics, whereby the rate of loss would be proportional to $\frac{P}{{K_{\text{M}} + P}}$, which is independent of $P$ so long as $P \gg K_{\text{M}}$ (where $K_{\text{M}}$ is the Michaelis constant for the reaction and dependent on enzymatic binding and dissociation rate constants). In general, such zeroth-order kinetics arises for many biochemical reactions whose rates are fit as Hill functions with rate proportional to $\frac{{P^{n} }}{{K_{\text{H}}^{n} + P^{n} }}$ with Hill coefficient $n$, so long as $P \gg K_{\text{H}}$.

Finally, the only accumulated quantity in a neuronal feedback system could be the number of receptors or channels in the membrane if the rate of removal of channels is saturated. For example, if proteins in the ubiquitination pathway are required to internalize and remove channels, and those proteins are in relatively short supply, then the rate of loss of channels could be independent of the total number of channels. In that case, the integrator in the feedback system would be at the final stage. In particular, when considering homeostasis of synaptic strength and homeostasis of intrinsic excitability, the two processes would unavoidably have different integrators and should be thought of as different controllers. The set-point of each type of channel/receptor would be established by its fixed rate of loss from the membrane (likely to be different for different channels/receptors in different parts of the cell) being matched by an activity-dependent rate of insertion.

**Fig. 5:**
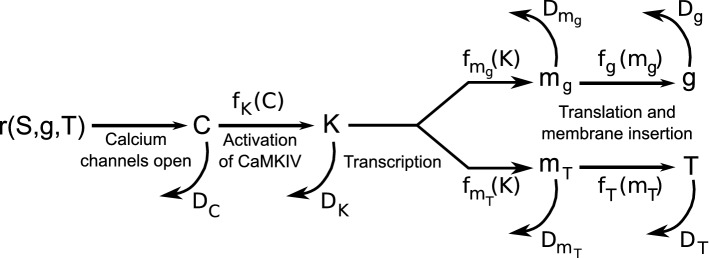
Structure of feedback pathways which lead to homeostatic changes in excitatory synaptic conductance, $g$, and firing threshold, $T$, for the neuron in response to changes in firing rate, $r$. Intermediate variables correspond to calcium concentration (Turrigiano 2008), $C$, CaMKIV activation (Joseph and Turrigiano 2017; Ibata et al. 2008; Goold and Nicoll 2010), $K$, and mRNA abundance (Ransdell et al. 2012), $m$. Equations 17–22 model this feedback scheme, based on the work of O’Leary et al (2013, 2014). Functions, $f$, represent forward rates (following horizontal arrows) and the functions, $D$, represent degradation rates, one of which should be independent of the degraded quantity

To understand the impact of the integrator being at these different points in the pathway, both on the system’s stability and its ability to respond to different perturbations of its inputs, we focused on a general model of the feedback pathways based on the work of O’Leary et al (2013, 2014). In this model, a set of biochemical pathways leads to modification of synaptic conductance, $g$, and threshold, $T$, in response to a neuron’s firing rate. The corresponding equations are:

17 $$ \frac{{{\text{d}}C}}{{{\text{d}}t}} = - \frac{C}{{\tau_{C} }} + r $$

18 $$ \tau_{K} \frac{{{\text{d}}K}}{{{\text{d}}t}} = - D_{K} \left( K \right) + f_{K} \left( C \right) $$

19 $$ \tau_{{m_{g} }} \frac{{{\text{d}}m_{g} }}{{{\text{d}}t}} = - D_{{m_{g} }} \left( {m_{g} } \right) + f_{{m_{g} }} \left( K \right) $$

20 $$ \tau_{{m_{T} }} \frac{{{\text{d}}m_{T} }}{{{\text{d}}t}} = - D_{{m_{T} }} \left( {m_{T} } \right) + f_{{m_{T} }} \left( K \right) $$

21 $$ \tau_{g} \frac{{{\text{d}}g}}{{{\text{d}}t}} = - D_{g} \left( g \right) + f_{g} \left( {m_{g} } \right) $$

22 $$ \tau_{T} \frac{{{\text{d}}T}}{{{\text{d}}t}} = - D_{T} \left( T \right) + f_{T} \left( {m_{T} } \right), $$

where we follow the scheme of Fig. 5 and have assumed calcium removal is linear in its concentration, $D_{C} \left( C \right) = - \frac{C}{{\tau_{C} }}$. In the following sections, we shall implement such a controller.

### The problem of filtering

As we have seen, a homeostatic control system requires components that can be stable at many different levels. For example, if a neuron is to raise its excitability (i.e., reduce its threshold) in response to a reduction in excitatory input it would need to adjust and then stably maintain a new density of sodium and/or potassium channels in its soma. Key to the production of such multi-stable components is an underlying process whose reverse rate does not depend on the accumulated state. In a biochemical system, this corresponds to a zeroth-order reaction rate, such as $D_{g} \left( g \right)$ in Eq. 21 and $D_{T} \left( T \right)$ in Eq. 22 being independent of $g$ and $T$ respectively.

Indeed, in a homeostatic control system, one might be able to identify the zeroth-order reaction as the point of integration and thus the controller in the system. To assess whether the system has a single homeostatic controller or two such controllers becomes a question of where in the feedback system—i.e., before or after a single feedback signal branches into two distinct signaling pathways—the point of integration arises. For example, at the extreme end, if integration occurs only at the point of channel insertion and removal—i.e., if $D_{g} \left( g \right)$ and $D_{T} \left( T \right)$ were constants—then the control of synaptic strength must be distinct from the control of intrinsic excitability.

However, the stability and utility of dual controllers are compromised if the integration step is too far removed from the initial signal. This is because at each step in the signaling pathway, the initially rapidly fluctuating firing-rate-dependent calcium signal is filtered so that the downstream fluctuations can be damped. Essentially, if rapid calcium fluctuations, varying on a timescale of 100 ms, are damped by processes which allow for variation on a timescale of 10 s, then the 100-fold increase in timescale causes a 10-fold reduction in the standard deviation (and thus a 100-fold reduction in the variance) of the downstream signal. Yet, as we saw in Sect. 2, both the benefit and the stability of a system with dual controllers rely upon setting a nonzero variance of the signal being controlled. If that signal is constrained to have low variance—by being a filtered version of a noisy signal—then the constraints on the controller’s set-points become too tight for wind-up to be avoided and/or the ability of the two controllers to control the firing rate variance (which has diminished impact on the signal they can control) is lost. Therefore, for dual control to be feasible, a method of maintaining a large variance in the input to the integrator is necessary. Such variance could be maintained by faithful transmission (without filtering) of the somatic calcium signal, which fluctuates rapidly according to the spike train. Alternatively, as we shall investigate in the next section, when filtering is present, nonlinearities in the signal processing pathway could boost the variance in the downstream signal.

### The benefit of nonlinearities

The reduction in variability by temporal filtering and resulting problems for a dual-control system can be ameliorated if the signaling pathways produce supralinear downstream responses. For example, introduction of a cubic or quartic nonlinearity can provide sufficient supralinearity of the response to allow a downstream signal to fluctuate a lot in spite of much temporal filtering of the upstream fluctuations in firing rate. Such a strong supralinearity is not unreasonable, since, for example, the very first step in most calcium signaling pathways is the binding of calcium by calmodulin, which is fully activated when bound to four calcium ions (so its activation can depend on up to the 4^th^ power of calcium concentration).

**Fig. 6:**
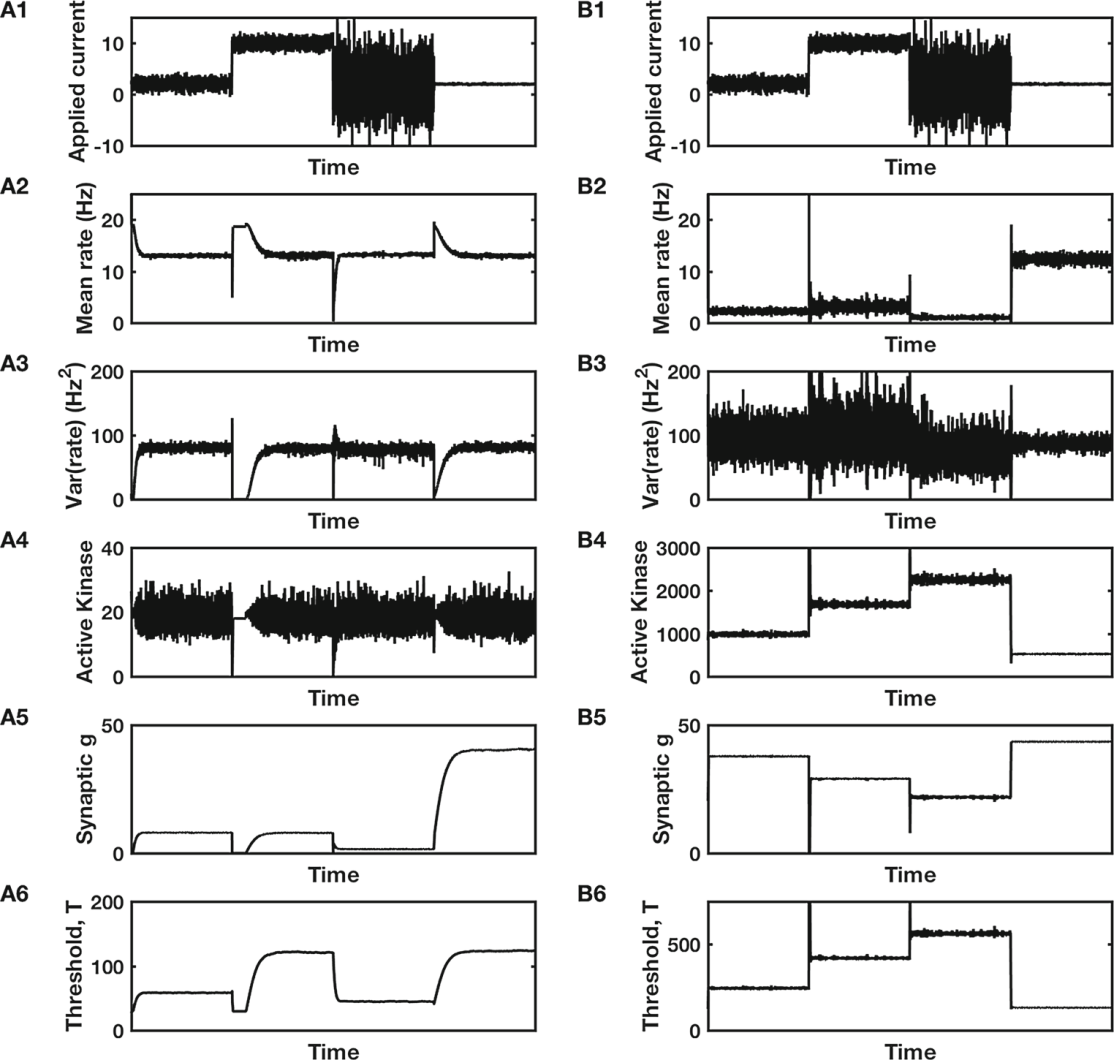
Homeostatic response of a biochemical pathway with **A** two controllers and **B** a single controller. Results of simulating the system depicted in Fig. 5 with integration produced via concentration-independent degradation in **A** at the mRNA/protein expression stage, or in **B** at the kinase-activation stage. **A1**, **B1** Input current with four different ranges. **A2**, **B2** Mean firing rate and **A3**, **B3** variance in firing rate in response to the inputs of **A1**, **B1**. Response of the single controller (**B**) is more rapid than that of the dual controller (**A**). In this example, since kinase activation is proportional to the cube of calcium concentration, it is the cube of the firing rate that is controlled, rather than mean rate itself. **A4, B4)** Kinase activation in the dual controller (**A**) tracks firing rate, but in the single controller (**B**) integrates rate so the stable levels of activation vary over a wide range. **A5**, **B5** Compensatory changes in synaptic conductance and **A6**, **B6**) intrinsic threshold of the neuron are co-regulated in a single controller (**B**) so that a rise in threshold (reduced excitability) always occurs with a reduction in synaptic conductance, whereas in the dual controller (**A**), synaptic conductance can increase when threshold increases (Maffei and Turrigiano 2008). Code used to produce this figure is available as intracellular_homeostasis.m

In Fig. 6A we see that inclusion of a cubic supralinearity allows for the homeostatic feedback system to regain stable control. [We use cubic rather than quartic, because an early fit to the activation of a similar calcium calmodulin-dependent kinase as a function of calcium suggested a coefficient of three (Abraham et al. 1996)]. The system is shown to adapt successfully to a 25-fold range of input variance as well as a 10-fold range of mean input current.

**Fig. 7:**
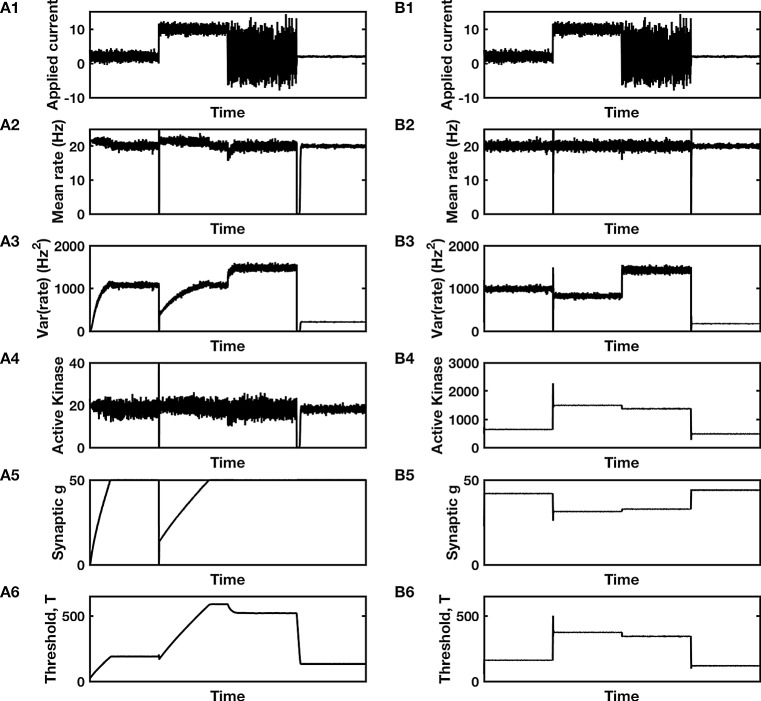
Compromised homeostatic response of a biochemical pathway with **A** two controllers and **B** a single controller. Results of simulating the system depicted in Figs. 5 and 6, but with linear activation of CaMKIV. Integration is produced via concentration-independent degradation in **A** at the mRNA/protein expression stage, or in **B** at the kinase-activation stage. **A1**, **B1** Input current with four different ranges. **A2**, **B2** Mean firing rate and **A3**, **B3** variance in firing rate in response to the inputs of **A1**, **B1**. Response of the single controller (**B**) is more rapid than that of the dual controller (**A**). In this example, since kinase activation is proportional to calcium concentration, the mean rate is well controlled; however, variance in firing rate is not controlled in either system. **A4**, **B4** Kinase activation in the dual controller (**A**) tracks firing rate, but in the single controller (**B**) integrates rate so the stable levels of activation vary over a wide range. **A5**, **B5** Compensatory changes in synaptic conductance and **A6**, **B6** intrinsic threshold. In the dual controller, panels A5-A6, indicate wind-up, where the synaptic conductance and threshold both increase until the synaptic conductance remains fixed at its maximum level. Code used to produce this figure is available as intracellular_homeostasis_lin.m

Such a dual system performs well over a broad range of parameters. For example, if we compare performance, as measured by the correlation between output rate and input current, of each controller, the dual controller with a cubic linearity betters the best single controller as a parameter such as $D_{g}$ is varied from its minimal value of $D_{T}^{2}$ (as necessary to ensure variance is positive) up to twice its minimal value. Beyond this range, wind-up impacts the dual controller’s performance negatively. However, if the supralinearity is absent, the dual controller is only optimal and able to avoid wind-up over a much smaller range, with an increase of 15% beyond the minimal value sufficient for wind-up to set in and render its performance worse than the single controller. Figure 7 demonstrates this point, with the value of $D_{g}$ set at approximately 25% greater than $D_{T}^{2}$ ($D_{g} = 400$, $D_{T} = 18$), the same as used in Fig. 6. The absence of the cubic linearity in CaMKIV activation leads to wind-up in the dual-control model.

## Summary and conclusions

In this article, we have considered how two control processes can cooperate rather than compete when they respond to the same signal, namely a single neuron’s firing rate. The presence of separate controllers for excitatory synaptic conductance and intrinsic excitability in neurons allows a neuron to produce outputs that vary across a desired range and to compensate for changes in both the variance and the mean of its inputs. However, when two controllers respond to the same variable, namely a neuron’s spike train, additional constraints are necessary to ensure the two controllers do not compete and produce “wind-up.” We have shown that the constraints include two essential features—the controlled variable should change rapidly compared to the timescale of the control process, and the controllers should respond nonlinearly to the controlled variable. Moreover, the controller that has a relatively greater impact on the gain of the system compared to the firing threshold should have a greater supralinearity in its response and a higher effective set-point.

If, instead, different controllers respond to distinct enough signals—for example, if one were to respond to synaptic input while the other responds to firing rate—then the system of distinct controllers would be more robust and competition between set-points avoided. An intriguing case arises if one control system is based on a single neuron’s firing rate while another monitors neighborhood activity. In such a situation, it is again possible for the controllers to compete since each neuron contributing to neighborhood activity also has its individual firing rate being controlled. Therefore, for the system to function, it would be important that neurons’ individual set-points are not incompatible with the set-point desired for the mean neighborhood activity. In such a situation, the issues addressed and techniques formulated in this article are relevant.

Whether a biochemical feedback system acts more as a single controller or a dual controller depends on whether the integration step occurs before or after two control pathways branch from each other. The point of integration can be determined by whether a variable in the system simply filters the output firing rate—so eventually returns to its prior level following compensation to a change in inputs—or integrates up the error and so remains at a distinct new level following a compensatory process. For example, somatic calcium concentration acts as a filter of spikes and indicates spike rate, so returns to its prior level following a period of disrupted activity which is then compensated for. On the other hand, conductance of excitatory synapses would remain elevated following such compensation. It will be of interest to assess whether activation of CaMKIV follows more the trajectory of internal calcium, or of synaptic conductance.

Therefore, to experimentally distinguish a dual-control system from a single control system with two outputs, it would be valuable to analyze measurements of the activation of CaMKIV both during and after homeostatic compensation from perturbations that change firing rates of neurons. If the change in firing rates causes a change in CaMKIV levels, but as firing rates return via cell-internal homeostatic mechanisms, so CaMKIV activation returns to baseline, then a dual-control mechanism is supported. However, if CaMKIV activation only shifts monotonically and remains shifted once the neuron returns to its prior activity patterns, then CaMKIV activation has the properties of an integrator for a single control system.

A second line of experiment that could weigh against a dual-control system would be observation of correlations between the abundance of mRNA for synaptic proteins such as those for AMPA receptor units, which undergo homeostatic regulation, and the abundance of mRNA for the proteins of homeostatically regulated intrinsic sodium or potassium channels. Such correlation would be expected in a system with a single control system in which transcription of these distinct mRNAs is co-regulated. For example, low firing rates would increase mRNA abundance for proteins associated with AMPA receptors and sodium channels while decreasing mRNA abundance for proteins associated with potassium channels. Observation of such correlation between mRNA abundances (Schulz et al. 2007; Tobin et al. 2009) as well as conductance values for different intrinsic channels (Goaillard et al. 2009) has previously provided strong evidence for a single control mechanism underlying these diverse intrinsic properties (O’Leary et al. 2013).

Intriguingly, in a dual-control system, it is possible to observe synaptic and intrinsic properties shifting in opposing direction—for example threshold could increase at the same time as excitatory synaptic conductance increases (Fig. 6A5, A6). Observation of such behavior would be evidence for a dual-control system if other plasticity mechanisms are absent or already accounted for.

Finally, data arising from protocols in which mean neural firing rate is maintained constant, but the statistical properties of the activity are altered, would provide valuable fodder to our efforts to understand the underlying control processes. For example, observations of how the neuron responds to alternating periods of quiescence and higher frequency firing while keeping mean rate fixed, as the durations of periods or the rate of high-frequency firing are altered, would be illuminating. Homeostasis arising from such protocols would not only support a theory of dual mechanisms but would constrain many of the underlying components of such a dual-control model.

## Note

MATLAB codes used to produce indicated figures are available at https://github.com/primon23/combined_homeostasis.
